# Local estrogen metabolism in epithelial ovarian cancer suggests novel targets for therapy

**DOI:** 10.1016/j.jsbmb.2015.03.010

**Published:** 2015-06

**Authors:** Xia Ren, Xuan Wu, Stephen G. Hillier, K. Scott Fegan, Hilary O.D. Critchley, J. Ian Mason, Sana Sarvi, Christopher R. Harlow

**Affiliations:** MRC Centre for Reproductive Health, The University of Edinburgh, The Queen’s Medical Research Institute, 47 Little France Crescent, Edinburgh, Scotland EH16 4TJ, United Kingdom

**Keywords:** Estrogen, Steroid sulfatase, Estrogen sulfotransferase, Epithelial ovarian cancer, Ovarian surface epithelium

## Abstract

Epithelial ovarian cancer (EOC) accounts for about 90% of malignant ovarian tumors, and estrogen is often implicated in disease progression. We therefore compared the potential for gating of estrogen action *via* pre-receptor metabolism in normal human ovarian surface epithelium (OSE), EOC and selected EOC cell lines (SKOV3 and PEO1). Steroid sulphatase (STS), estrogen sulfotransferase (EST), 17β-hydroxysteroid dehydrogenases 2 (17BHSD2) and 5 (17BHSD5) mRNAs, proteins and enzymatic activities were all detectable in primary cell cultures of OSE and EOC, whereas aromatase and 17BHSD1 expression was negligible. qRT-PCR assay on total mRNA revealed significantly higher EST mRNA expression in OSE compared to EOC (*P* < 0.05). Radioenzymatic measurements confirmed reduced sulfoconjugation (neutralization) of free estrogen in EOC relative to OSE. OSE cells were more effective at converting free [^3^H]-E_1_ to [^3^H]-E_1_S or [^3^H]-E_2_S, while EOC cell lines mainly converted [^3^H]-E_1_ to [^3^H]-E_2_ with minimal formation of [^3^H]-E_1_S or [^3^H]-E_2_S. IL1α treatment suppressed EST (*P* < 0.01) and 17BHSD2 (*P *< 0.001) mRNA levels in OSE and stimulated STS mRNA levels (*P* < 0.001) in cancer (SKOV3) cells. These results show that estrogen is differentially metabolized in OSE and EOC cells, with E_2_ ‘activation’ from conjugated estrogen predominating in EOC. Inflammatory cytokines may further augment the local production of E_2_ by stimulating STS and suppressing EST. We conclude that local estrogen metabolism may be a target for EOC treatment.

## Highlights

• Human ovarian surface epithelium (OSE) and epithelial ovarian cancer (EOC) cells have the capacity for local metabolism of estrogen.
• Estrogen is differentially metabolized in OSE and EOC cells, with E_2_ formation from conjugated estrogen predominating in cancer cells.
• Inflammatory cytokines augment the local production of E_2_ by stimulating steroid sulfatase and suppressing estrogen sulfotransferase.
• STS inhibition and/or EST augmentation (local estrogen metabolism) show promise as a target for EOC treatment.

## Graphical abstract

**Figure:**
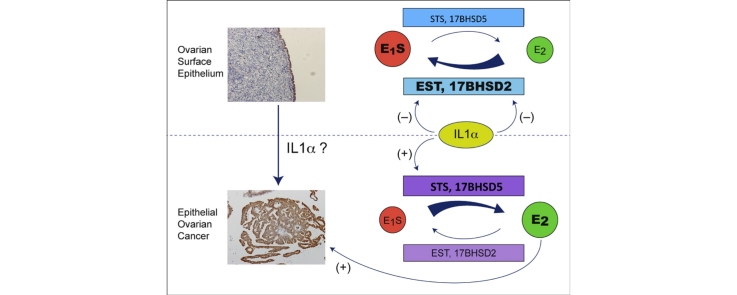

## 1 Introduction

Estrogen is implicated in the progression of ovarian cancer, which is the most lethal of all gynecological malignancies. Epithelial ovarian cancer accounts for about 90% of malignant ovarian tumors [1]. Epidemiological data are suggestive that estrogen-only hormone replacement treatment (HRT) users have a higher risk of ovarian cancer [2,3]. In addition, anti-estrogen intervention inhibits the growth of ovarian carcinoma *in vitro* and *in vivo* [4,5]. Furthermore, clinical trials proved the aromatase inhibitor letrozole to benefit a sub-group of ovarian cancer patients [6,7].

Estrogen action in most cells is transduced by the nuclear estrogen receptor (ER) isoforms ERα and/or ERβ. Most ovarian cancers are ER positive [8]. ERα predominates in EOC, whereas ERβ expression is higher in normal ovarian surface epithelium (OSE) [9]. Thus EOC is likely estrogen-responsive. Paradoxically, ovarian cancer generally occurs in post-menopausal women when the ovary no longer actively secretes estrogen. This raises the question: if estrogen is involved, how is it produced?

Many tissues in the body that are incapable of *de novo* estrogen biosynthesis can still generate estrogen through the hydrolysis of sulfoconjugated steroids reaching them from blood. Free E_2,_ capable of activating ER signaling can be formed from circulating E_1_S through the hydrolytic activity of STS and the 17-oxoreductase activity of 17BHSD5. Conversely, the oxidative function of 17BHSD2 produces the weak estrogen E_1_ from E_2_ and EST can sulfoconjugate E_1_ to further minimize estrogen action. Intracellular steroid activation through the STS pathway is involved in estrogen-dependent epithelial cancers, such as breast and endometrial carcinomas [10], and single nucleotide polymorphisms in SULT1E1 lead to increased risk of breast [11] and endometrial [12] cancers, together with reduced survival. A study of Jewish women predisposed to breast and ovarian cancer found a link to a missense mutation (His224Gln) in the SULT1E1 gene [13]. Together, these observations suggest that if these mutations affected enzyme activity, they might be candidates for cancer promotion. Furthermore, the already substantial levels of E_1_S that circulate in postmenopausal women are increased by hormone replacement therapy (HRT) [14].

We therefore hypothesize that E_2_, is produced locally from circulating E_1_S *via* the STS pathway in EOC cells. Additionally, since inflammatory cytokines such as IL1α secreted by OSE [15] are implicated in oncogenesis [16], they could have a role in activating estrogen formation within ovarian tumors. Here we demonstrate that EOC and normal OSE cells do indeed have distinct estrogen metabolizing signatures compatible with increased local generation of estrogen in ovarian cancer.

## 2 Materials and methods

### 2.1 Ovarian tissues

Non-pathalogical ovarian tissue was donated by pre-menopausal patients undergoing surgery for benign gynecological conditions (see Supplementary Tables 1 and 2 for clinicopathological information). None of the patients had evidence of endometriosis, nor did the OSE show any evidence of endometriotic lesions. Samples of ovarian cancer tissue were donated by 12 patients with confirmed ovarian cancer (see Supplementary Table 3 for clinicopathalogical details of ovarian cancer patients). Paraffin-embedded (non-pathalogical pre-menopausal, post-menopausal and cancerous) tissue from other patients was kindly arranged by Dr. Alistair A. Williams (Department of Pathology, University of Edinburgh). Formal written consent was obtained from all patients and the project approved by the Local Research Ethics Committee (COREG reference 04/S1103/36). Previously-characterized ER positive cell lines were SKOV3 (European Collection of Cell Cultures, Public Health England, Salisbury, UK) and PEO1 [17].

### 2.2 Cell collection and culture

OSE cells were collected by gently brushing the ovarian surface with a Tao brush (Cook Ireland Ltd., Limerick, Ireland) and rinsing OSE cells into T75 flasks (Corning Inc., Corning, NY) with culture medium (see below) as previously described [18,19]. Primary EOC cells were retrieved from ovarian cancer tissues by enzymatic dispersion [20]. In brief, tissue was minced with scalpel blades and incubated overnight at 4 °C in 0.25% trypsin (Gibco, Life Technologies, Paisley, Scotland), 0.004% DNAse1 (Sigma, Poole, Dorset, UK). Trypsin was inactivated with addition of serum-containing medium (see below) and the cells pelleted by centrifugation (500 × *g*, 5 min) before resuspension in fresh medium and culture to confluence in T75 flasks. The culture medium was Medium 199 (Gibco):MCDB 105 (Sigma) (1:1 v/v) containing 15% (v/v) fetal bovine serum (FBS), 50 μg/ml streptomycin, 50 IU/ml penicillin and 2 mmol/l l-glutamine (all from Sigma). OSE cells were used in experiments within two passages of culture since collection. SKOV3 and PEO1 cell lines were maintained in T162 flasks (Corning) in the same culture medium containing 10% (v/v) FBS.

### 2.3 Experimental treatments

Cells were plated into 6-well culture dishes at densities of 3 × 10^5^ cells per well for mRNA studies or 5 × 10^5^ cells per well for enzyme activity assays. Incubation was at 37 °C in a humidified atmosphere of air and CO_2_ (95:5 v/v). Cell monolayers were established by 24 h whereupon medium was substituted with serum-free medium containing 0.01% bovine serum albumin (BSA, Fraction V, Sigma) for a further 24 h. Experiments were then started by adding 0.5 ml fresh serum-free medium. IL1α (R & D Systems, Abingdon, UK) was used at a concentration of 0.5 ng/ml unless dose was a variable. Recombinant human IL1 receptor antagonist (IL1RA, R & D systems) was used at 25 ng/ml. Incubation was for 48 h for mRNA expression studies or 72 h for enzymatic studies, in which case radiolabeled substrate was also added to the medium (see below).

### 2.4 RNA extraction

Total RNA was extracted from washed cell monolayers using RNeasy Mini kit (Qiagen Ltd., Crawley, W Sussex, UK) as per the manufacturer’s protocol with on-column DNAse treatment. RNA concentration was measured using a Nanodrop spectrophotometer (ND-1000, Nanodrop Technologies Inc., Wilmington, DE, USA). RNA samples with 260/280 ratio above 2.0 were used for cDNA synthesis.

### 2.5 Quantitative Taqman real-time PCR analysis

Total RNA (200 ng) was reverse transcribed to cDNA using the RT-Reagent Kit (Applied Biosystems, Warrington, UK) according to the manufacturer’s protocol. Quantitative real-time PCR was performed in a reaction mixture containing 2 μl of cDNA, Taqman Universal PCR mastermix (Applied Biosystem) and specific primer/probe sets. STS and 17BHSD5 primer/probe sets were pre-validated (Assay-on-demand, Applied Biosystems); EST primer and probes were designed in-house using ProbeFinder version 2.45 (Roche Diagnostics Ltd., Burgess Hill, UK) and synthesized by Genosys Biotechnologies (Cambridge, UK). Primer and probes for 17BHSD2 were designed in-house by Primer Express software and manufactured by Biosource (Nivelles, Belgium). Sequences of primer/probe sets for qRT-PCR are listed in Table 1. Probes and primers were all validated before use. A ribosomal 18S primer/probe set (Applied Biosystems) was also included and used as an internal control. Target mRNA was quantified in relation to 18S rRNA in each sample. The negative controls comprised RT-negative (RNA reaction with no reverse transcriptase enzyme), RT-H_2_O (water in place of RNA) and a Taqman reaction-negative control (water instead of cDNA). Reactions were carried out in duplicate. Samples were evaluated in 96-well plates using an ABI Prism 7900 Sequence Detector (Applied Biosystem).

### 2.6 Enzyme activity assay

Enzyme activities were determined by radioenzymatic activity assays. The substrate was E_1_S or E_1_ (3 nM) including 150,000 cpm [6,7-^3^H(N)]-E_1_S or [2,4,6,7-^3^H]-E_1_ (PerkinElmer, USA) in 2 ml serum-free culture medium. Cells were incubated with or without IL1α at 0.5 ng/ml for 72 h. The media were collected and mixed with 10 ml dichloromethane (Fisher Scientific, Loughborough, UK) to stop the reaction. Samples were centrifuged and the organic phase removed and evaporated to dryness under nitrogen. The dried steroid extract was reconstituted in 100 μl dichloromethane with unlabeled E_1_ (10 μM), E_2_ (10 μM) and E_1_S (10 μM) as carrier. Samples were applied onto silica-gel pre-coated sheets (PE, SILG; Whatman, Maidstone, Kent, UK) and thin-layer chromatography undertaken using chloroform:ethanol (92:8, v/v) as the mobile phase. Radio-labeled steroid components were identified and quantified using a Bioscan 200 imaging detector (Lablogic Systems, Sheffield, UK).

### 2.7 Immunohistochemistry

Immunohistochemistry was done on 10 μm thick paraffin-embedded tissue sections, Standard protocols were used involving antigen retrieval in 0.01 M citrate buffer (pH 6.0), blockade of endogenous peroxidase and streptavidin-biotin, and a non-immune serum blocking step. Slides were incubated overnight at 4 °C with primary antibody (rabbit anti-human STS polyclonal antibody (Sigma–Aldrich, HPA002904), 1:75; rabbit anti-mouse EST polyclonal antibody [21], 1:750; rabbit anti-human 17BHSD2 polyclonal antibody [22], 1:200; mouse anti-human 17BHSD5 monoclonal antibody [23], 1:200; mouse anti-human aromatase monoclonal antibody [24], 1:50) followed by incubation with secondary antibody (goat anti-rabbit 1:500 for STS, EST and 17BHSD2, goat anti-mouse 1:500 for 17BHSD5 and aromatase, Dako, Cambridge, UK) at room temperature for 60 min. Streptavidin horseradish peroxidase (Vector, Peterborough, UK) was used to amplify the signal from the biotinylated secondary antibody and specific immunostaining was visualized by 3,3-diaminobenzidine (DAB; Dako, Cambridge, UK). Generic immunoglobulins from the same species or green fluorescent protein (GFP, Invitrogen, Paisley, UK) antibody raised in the same species at the same concentration as the primary antibody were used as negative controls. Positive controls were tissue sections from placenta for STS and 17BHSD2, fetal kidney for EST and endometrium for 17BHSD5. Photomicrographs were taken using a Provis AX70 microscope (Olympus) with ×10 and ×20 objectives, and an Axiocam (Carl Zeiss) digital camera with Axiovision image capture software (Carl Zeiss).

### 2.8 Statistical analysis

Statistical analysis was performed using GraphPad Prism 5 (GraphPad Software Inc., San Diego, USA). qRT-PCR data were analyzed by the Mann–Whitney test (Fig. 2), one-way analysis of variance (ANOVA, Fig. 5A and C), two-way ANOVA (Fig. 5B) and Wilcoxon signed rank test (Fig. 6). Enzyme activity assay data were analyzed by one-way ANOVA (Fig. 4). Statistical difference was assigned at *P *< 0.05. All post-hoc testing subsequent to ANOVA was by Tukey’s multiple comparisons.

## 3 Results

### 3.1 STS, EST, 17BHSD2, 17BHSD5 and aromatase protein expression

**Fig. 1:**
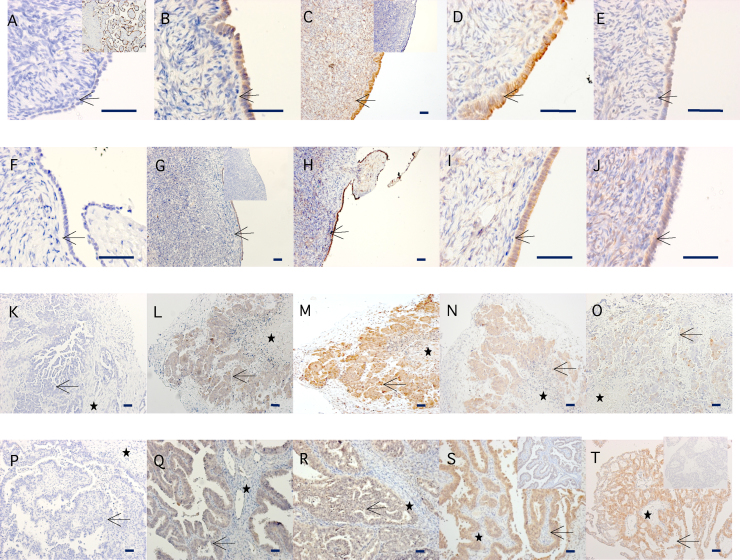
Immunolocalization of Aromatase (A, F, K, P), STS (B, G, L, Q), EST (C, H, M, R), 17BHSD2 (D, I, N, S) and 17BHSD5 (E, J, O, T) proteins in pre-menopausal (A–E), post-menopausal (F–J) ovaries and epithelial ovarian cancer (K–T). Expression was localized using specific antibodies raised against Aromatase, STS, EST, 17BHSD2 and 17BHSD5, as described in Section 2. Examples shown are representative of 3 pre-menopausal, 6 post-menopausal and 7 EOC patient samples. The clinicopathalogical profiles of the samples are given in Supplementary Tables 1 and 3. (K–O) Aromatase, STS, EST, 17BHSD2 and 17BHSD5 in high grade serous carcinoma, (P) Aromatase in endometrioid carcinoma grade 3, (Q) STS in a mixed high grade serous and endometrioid carcinoma, (R) EST in endometrioid carcinoma grade 3, (S) 17BHSD2 in endometrioid carcinoma grade 3, (T) 17BHSD5 in mixed high grade serous and endometrioid carcinoma. Arrow indicates OSE cells (A–J) or epithelial-like cells (K–T). Asterisk marks stromal tissue. Inserts show positive control-placenta (A) or non-immune serum controls (C, G, S, T). Scale bars 40 μm.

STS, EST, 17BHSD2 and 17BHSD5 were readily detected in pre-menopausal and persists in post-menopausal ovaries and EOC (Fig. 1). Positive immunostaining was particularly evident in the OSE layer of normal tissues and the lining of EOC lesions. Aromatase expression was undetectable in the OSE of normal pre- or post-menopausal ovaries or EOC, despite strong expression in positive control (placenta) tissue.

### 3.2 STS, EST, 17BHSD1, 17BHSD2, 17BHSD5 and aromatase mRNA expression

**Fig. 2:**
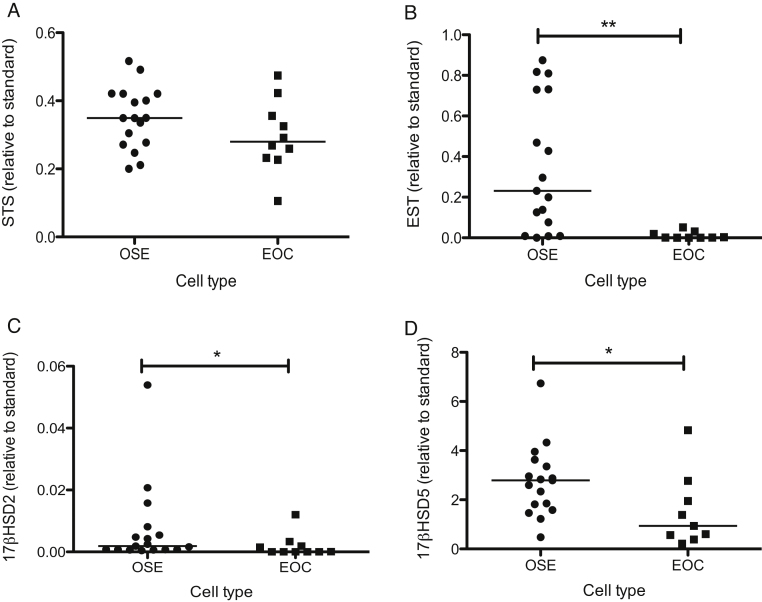
Expression of STS (A), EST (B), 17BHSD2 (C) and 17BHSD5 (D) mRNAs in OSE (*n *= 17) and EOC (*n *= 9–10). RNA was extracted from untreated cells and measured using qRT-PCR. The level of mRNA was standardized to 18S rRNA and presented as fold-increase relative to standard (placenta for STS and 17BHSD5, endometrium for EST and 17BHSD2). Horizontal bars indicate median value (*P < 0.05, **P < 0.01, Mann–Whitney test).

STS mRNA was measurable at similar levels in OSE and EOC (Fig. 2A). EST mRNA expression was significantly higher in OSE than EOC (Fig. 2B, *P *< 0.01). 17BHSD2 mRNA was expressed in all OSE and most EOC samples. However, the median level in OSE was slightly higher than that in EOC (Fig. 2C, *P* < 0.05). 17BHSD5 mRNA was also higher in OSE than EOC (Fig. 2D, *P *< 0.05). 17BHSD1 and aromatase mRNA expression were almost undetectable (less than 10,000-fold compared to placenta standard – results not shown).

### 3.3 Estrogen receptor (ER) mRNA expression

**Fig. 3:**
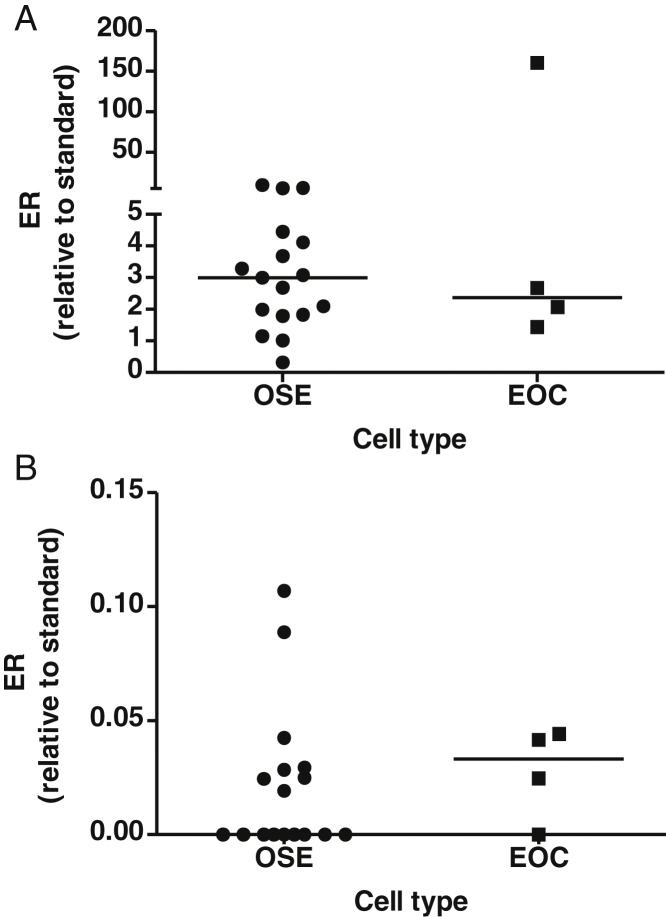
Expression of ERα (A) and ERβ (B) mRNA expression in OSE (*n* = 17) and EOC (*n* = 4) by Taqman qRT-PCR. RNA was extracted from untreated cells and measured using qRT-PCR. The level of mRNA was standardized to 18S rRNA and presented as fold-increase relative to standard (placenta). Horizontal bars indicate median value.

ERα and ERβ mRNA expression were determined in OSE cells and a subset of EOC (Fig. 3). ERα was expression was not significantly different between OSE and EOC (Fig. 3A). ERβ was detected in half of the OSE samples and 3 out of 4 EOC samples, and was not significantly different (Fig. 3B).

### 3.4 Estrogen metabolism

**Fig. 4:**
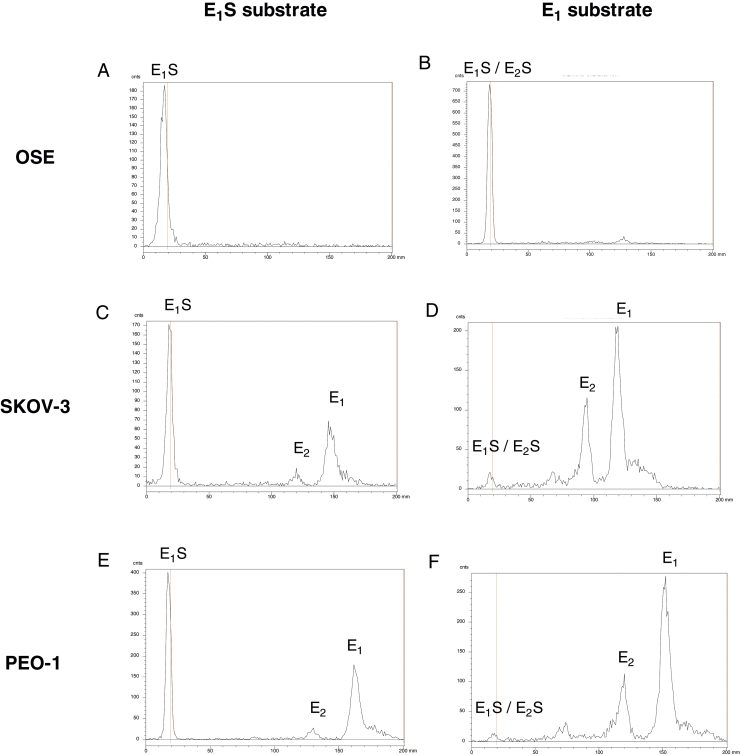
Radioenzymatic analysis of estrogen metabolism in OSE and cancer cell lines. Medium from cells incubated with radiolabeled substrate was extracted, chromatographed to separate substrate and metabolites, and scanned as described in Section 2. A–F, representative radiochromatographs from OSE (A, B), SKOV3 (C, D) and PEO1 (E, F) cells cultured for 72 h in presence of [^3^H]-E_1_S (A, C, E) and [^3^H]-E_1_ (B, D, F). Tritium labeled metabolites are shown as radioactive counts per minute plotted against distance along the plate (mm).

OSE cells did not measurably convert [^3^H]-E_1_S into free estrogen (Fig. 4A) or convert [^3^H]-E_1_ into [^3^H]-E_2_ (Fig. 4B). However, they efficiently conjugated [^3^H]-E_1_ to form [^3^H]-E_1_S (Fig. 4B).

In contrast two EOC cell lines readily produced free E_1_ and E_2_ from [^3^H]-E_1_S (Fig. 4C and E) and were effectively unable to sulfoconjugate [^3^H]-E_1_. Instead, when [^3^H]-E_1_ was the substrate, EOC cells mainly produced [^3^H]-E_2_.

**Fig. 5:**
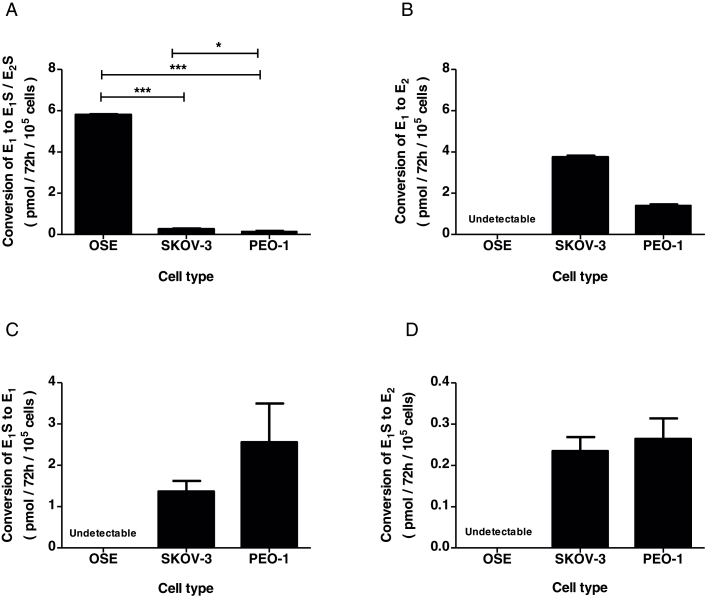
Aggregate estrogen metabolism profiles for OSE cells and cancer cell lines expressed as the net conversion of substrate to product (black columns). (A) EST and 17BHSD reductase, (B) 17BHSD reductase, (C) STS, (D) STS and 17BHSD reductase. Bars indicate mean ± SEM. (A) and (B) *n *= 3 (OSE, SKOV3 and PEO1); (C) and (D) *n *= 10 (OSE), *n* = 3 (SKOV3 and PEO1). Statistical analysis was by one-way ANOVA followed by Tukey’s post-hoc tests. (A) *P *< 0.001, *F *= 13,800, df = 2, 6. *P < 0.05, ***P < 0.001. Statistical analysis was not performed in B–D due to undetectable activity in OSE cells.

Quantification of the data confirmed that OSE cells have significantly higher estrogen sulfoconjugation potential compared to EOC lines (Fig. 5A) and substantially lower potential to activate E_1_ into E_2_ (Fig. 5B). On the other hand, EOC are significantly more able than OSE to produce free E_1_ (Fig. 5C) and E_2_ (Fig. 5D).

### 3.5 Regulation of STS mRNA by IL1α

**Fig. 6:**
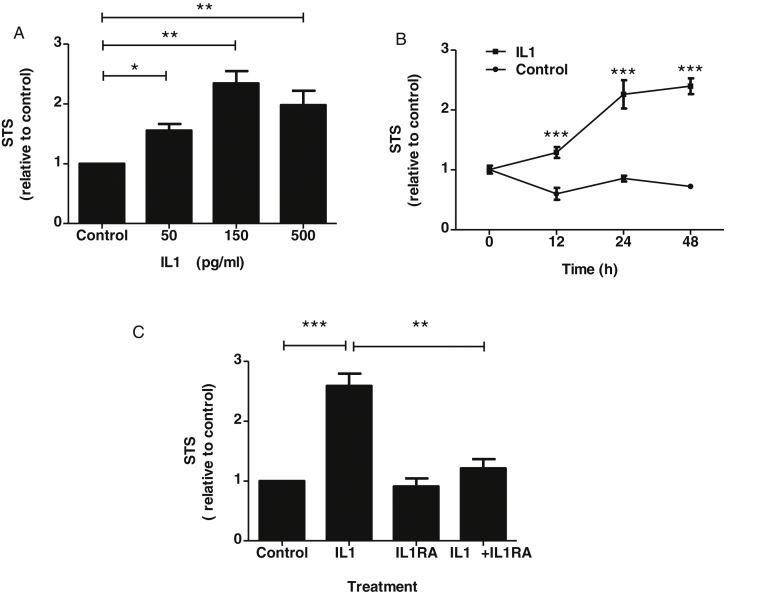
Regulation of STS mRNA by IL1α in SKOV3 cells. (A) Dose-dependent effect of IL1α. Cells were treated with increasing concentrations of IL1α for 48 h. Levels of mRNA were standardized to 18S rRNA and presented as fold increase relative to the positive control at each dose point (*P *< 0.01, *F *= 18.67, df = 3, 2, one-way ANOVA with Tukey’s post-hoc tests. * = *P* < 0.05; ** = *P *< 0.01). (B) Time-dependent effect of IL1α. Cells were treated with 0.5 ng/ml IL1α for times indicated. Levels of mRNA were standardized to 18S rRNA and presented as fold-increase relative to the corresponding untreated control at each time point (*P *< 0.01, *F *= 69.15, df = 1, 12, two-way ANOVA with Tukey’s post-hoc tests *** = *P* < 0.001). (C) Inhibition of IL1α-stimulated STS mRNA expression by IL1RA. Cells were cultured for 48 h in absence and presence of IL1α (0.5 ng/ml), without or with IL1RA (25 ng/ml). Total RNA was standardized to 18S rRNA and presented as fold increase relative to untreated control (*P *< 0.001, *F *= 36.64, df = 3, 2, one-way ANOVA with Tukey’s post-hoc tests **P < 0.01; ***P < 0.001). Bars and data points indicate mean ± SEM of 3 independent experiments.

The effect of an inflammatory cytokine on STS mRNA level as a proxy for steroid sulfatase activity potential was assessed in an EOC cell line (SKOV3). Treatment with IL1α caused time- (Fig. 6A) and dose-dependent (Fig. 6B) increases in STS mRNA which were fully prevented by the presence of IL1 receptor antagonist (IL1RA) (Fig. 6C, *P* < 0.01).

### 3.6 IL1α regulates estrogen-metabolizing enzyme mRNAs in OSE

**Fig. 7:**
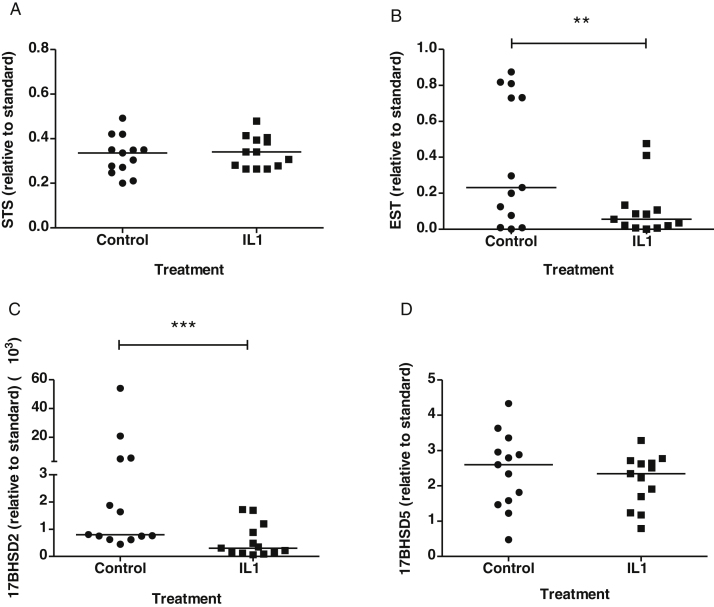
Regulation of STS (A), EST (B), 17BHSD2 (C) and 17BHSD5 (D) mRNAs by IL1α in OSE. Cultured OSE cells were treated with and without 0.5 ng/ml IL1α for 48 h, and total RNA was extracted for qRT-PCR analysis. Combined results were from 13 OSE. Level of mRNA was standardized to 18S rRNA and presented as fold increase relative to standard (placenta for STS and 17BHSD5, endometrium for EST and 17BHSD2). Horizontal bars indicate median value (**P < 0.01; ***P < 0.005, Wilcoxon signed rank test).

Treatment of OSE cells with IL1α for 48 h did not affect STS mRNA expression (Fig. 7A), but significantly decreased EST mRNA (Fig. 7B, *P* < 0.01) and 17BHSD2 mRNA (Fig. 7C, *P* < 0.001). 17BHSD5 mRNA expression was not affected by IL1α treatment (Fig. 7D).

## 4 Discussion

These results are suggestive that the key to estrogen generation in EOC cells may lie in their relative ability to convert conjugated estrogen substrates into free biologically active estrogens. Ovarian capacity to produce estrogen through aromatisation of androgens subsides after the menopause when folliculogenesis ceases. Here we show for the first time that increased ratios of STS/EST in EOC may facilitate local active estrogen synthesis in EOC and that inflammatory cytokines may trigger this synthesis in OSE. Circulating E_1_S in post-menopausal women is around 0.4 nM and increases to about 7 nM in women who are taking HRT [25], indicating sufficient substrate in circulation for significant local E_2_ production in post-menopausal ovarian cancer patients.

Our data complement previous studies showing expression and activity of STS in ovarian cancer cells [26,27], with evidence for a negative correlation between sulfatase activity and progression – free survival in patients with advanced stage epithelial ovarian cancer [28]. We now add substantially to these observations by comparing the expression of genes encoding pre-receptor metabolism and production of estrogen in normal OSE and EOC. Importantly we find STS mRNA and protein expression in both OSE and EOC cells, as well as in SKOV3 and PEO1 cell lines, confirming the potential for OSE and EOC to generate free estrogen *via* hydrolysis of circulating E_1_S. This complements evidence for estrogen generation from sulfated forms in breast cancer tissue, where sulfatase pathway is 50–200 times more active than aromatase [29]. The additional presence of 17BHSD5 mRNA and protein in both OSE and EOC cells further indicates the possibility of E_2_ production from E_1_. The persistence of expression of STS, EST and 17BHSD2/5 in post-menopausal ovarian OSE indicates that enzymatic potential remains, even after cessation of follicular activity in the ovary.

Conversely, the presence of EST and 17BHSD2 in OSE and EOC lends potential to the deactivation free estrogen through reverse metabolism of E_2_ to E_1_ and sulfoconjugation into E_1_S. Thus among other things, the estrogen-generating potential would seem to depend on the balance of STS/17BHSD5 *versus* EST/17BHSD2. We find STS mRNA expression to be similar in OSE and EOC cells whereas EST mRNA expression is substantially increased in OSE. Furthermore, 17BHSD2 mRNA levels are substantially lower in OSE compared with EOC while differences in 17BHSD5 mRNA levels are much less. These results are also in broad agreement with a recent microarray study on 12 samples of ovarian cancer epithelial cells and 12 samples of normal OSE [30], in which STS and 17BHSD1 (an alternative 17-oxoreductase to 17BHSD5) were higher, but 17BHSD2 was lower, in EOC compared with OSE. The mRNA expression profiles in both studies imply a bias toward active estrogen formation in EOC relative to OSE.

Whilst expression of aromatase in granulosa cells is universally recognized, the expression in OSE is less clear. We were unable to detect immunohistochemical localization of aromatase in single-layered OSE. This was in contrast to positive immunostaining reported previously [31], although the multi-layered OSE did not resemble that observed in the present study. Furthermore, aromatase mRNA expression in OSE was 10,000-fold lower than placental tissue, suggesting that presence of aromatase transcripts in these cells is negligible. Evidence for aromatase expression in EOC and cancer cell lines is more compelling, although not universally demonstrated in all cases or in all studies [32–35]. Interestingly, aromatase expression was noted in stromal cells, but not carcinomatous epithelial cell nests [36]. The lack of aromatase immunohistochemical or mRNA expression in the present study may be a consequence of the relatively small number of samples studied.

The potential for increased estrogen formation in EOC is verified by our measurements of estrogen (in) activation in vitro. Thus OSE cells tend to produce more conjugated estrogens from free estrogen substrates whereas EOC cells more readily mobilise estrogen from conjugated substrates. These data therefore suggest that relative protection from biologically active estrogen in OSE is lost in EOC.

Further mechanistic insight into the role of altered estrogen metabolism in the etiology of EOC will require the use of suitable transgenic EOC mouse models. *Sult1e1* mRNA was observed in the OSE of mouse ovaries, and a global *sult1e1* knockout mouse had impaired ovulation [37]. EST protein expression was downregulated in older mice [38], and mouse OSE can undergo transformation *in vitro* and form tumors after i.p. injection into syngenic and athymic recipients [39]. Furthermore, in a mouse model of ovarian cancer, tumorigenesis was dependent on local estrogen production within the tumor [40]. A definitive investigation of the protective role of EST will likely require the use of a conditional *sult1e1* knockout mouse, but this limited animal data support our observations.

Similar ERα and ERβ mRNA expression have been described previously in OSE and EOC [41]. In contrast, Brandenberger et al. [42] using Northern analysis, and Bardin et al. [43] using qRT-PCR found lower levels of ERβ mRNA in EOC compared with OSE, that was not mirrored in the present study. Although there remains debate as to which ER type is more important in estrogen action in EOC, our data support a role of ER-mediated action of locally produced estrogen in both normal OSE and EOC.

Our data also indicate that the machinery for producing active estrogen from inactive conjugated estrogens in OSE is susceptible to inflammatory stimulation in normal and cancer cells, demonstrating a potential mechanism of tumor progression in EOC.

In OSE cells, treatment with IL1α had no effect on STS and 17BHSD5 mRNA levels while it decreased in EST and 17BHSD2 mRNA expression in response to IL1α. This implies a net stimulatory effect of IL1α on the potential for active estrogen formation by OSE. Conversely, IL1α markedly increased STS expression - hence potential - for production of E_1_ in SKOV3 cell line. The SKOV3 data suggests an inflammatory basis for aggravation of EOC *via* local hydrolysis of E_1_S. There is evidence for induction of STS activity by other inflammatory cytokines (IL6 and TNFα) in breast cancer *via* a post-translational modification of the enzyme or by increasing substrate availability [44]. It remains to be determined whether TNFα and IL6 increase STS activity in ovarian cancer cells. IL1β suppressed STS mRNA and activity in endometrial stromal cells [45], although this may reflect tissue specific different cytokine actions on epithelial and stromal cells.

The finding that the STS pathway could contribute to the progression of estrogen-dependent ovarian cancer highlights the potential importance of STS as a therapeutic target in the treatment of gynecologic cancer. Recent evidence points to successful use of an STS inhibitor in other cancers. In a hormone-dependent endometrial xenograft model using ovariectomized mice, the STS inhibitor STX64 reduced tumor growth by 48% [46]. A phase 1 study of STX64 in breast cancer patients showed good tolerance, inhibition of STS activity in tumor tissues and a significant decrease in circulating estrogenic steroid concentration [25]. STS activity was blocked by STX64 in the ovarian cancer cell line OVCAR-3 [47]. Collectively, these data emphasize the therapeutic potential that STS inhibition might hold for the treatment of ovarian cancer. Conversely, EST might be augmented to the same end.

In conclusion, we present novel evidence that estrogen intracrinology differs substantially in OSE and EOC cells. Our study suggests a mechanism through which E_2_ formation could be suppressed in OSE and augmented in EOC through differential metabolism of free and conjugated estrogen substrates, mediated by binding to ER. Their metabolic profiles imply reduced sulfoconjugation and inactivation of estrogen in EOC through reduced STS and/or enhanced EST activities. Inflammatory cytokines potentially exacerbate these differences. Further studies are required to fully understand the mechanisms involved. Targeted STS inhibition and/or EST augmentation present strategies for manipulating steroid-responsive cancer cell growth *in vitro* and *in vivo*, and may ultimately lead to the development of more effective treatments for ovarian cancer.

## Conflict of interest

The authors declare that there is no conflict of interest that could be prejudicial to the impartiality of the research reported.

## Funding

This work was supported by the Medical Research Council (grant numbers G0500047 and G0900550).

## Author contributions

X. Ren, X. Wu, and S. Sarvi conducted the experiments. X. Ren, S.G. Hillier and C.R. Harlow wrote the manuscript. K.S. Fegan and H.O.D. Critchley collected the clinical samples. J.I. Mason provided valuable editorial commentary.

## Figures and Tables

**Table 1:** Sequences of primer/probe sets for qRT-PCR. Sequences of assay-on-demand primers and probes were unavailable but were pre-validated by ABI. Probe number referred to the number of the probe in the Universal Probe Library and sequences were unavailable.

Gene	Forward primer (5′-3′)	Reverse primer (5′-3′)	Probe (5′FAM-TAMRA/MGB 3′)	NCBI accession or reference number
Aromatase	Assay-on-demand	Assay-on-demand	Assay-on-demand	Hs00240671_m1
*STS*	Assay-on-demand	Assay-on-demand	Assay-on-demand	Hs00165853_m1
*EST*	AAGACTCATTTGCCACCTGAA	GCATTCCGGCAAAGATAGAT	Roche Probe library number: 4	NM_005420.2
*HSD17B1*	Assay-on-demand	Assay-on-demand	Assay-on-demand	Hs00166219_m1
*HSD17B2*	TGTCAGCAGCATGGGAGGA	GGTCACAGCCGCCTTTGAT	CCCCAATGGAAAGGCTGGCATCTT	NM_002153.2
*HSD17B5*	Assay-on-demand	Assay-on-demand	Assay-on-demand	Hs00366267_m1
*ERα*	TGATTGGTCTCGTCTGGCG	CATGCCCTCTACACATTTTCCC	TGCTCCTAACTTGCTCTTGGACAGGAACC	NM_000125.3
*ERβ*	GGTCCATCGCCAGTTATCACAT	GATGCGTAATCGCTGCAGACAG	TGTGAAGCAAGATCGCTAGAACACACCT	NM_001437.2
