# Supplementary material for: Local estrogen metabolism in epithelial ovarian cancer suggests novel targets for therapy
Source: J Steroid Biochem Mol Biol. 2015 Jun;150:54–63. doi: 10.1016/j.jsbmb.2015.03.010 (PMC4429663; doi:10.1016/j.jsbmb.2015.03.010)
Supplement: Supplementary file 1 [file mmc1.docx]

| **Patient /block**  **number** | **Age (years)** | **Reason for surgery** | **Ovary diseases** | **Pre/post menopausal** |
| --- | --- | --- | --- | --- |
| 1 | 47 | Fibroids | No | Pre-menopausal |
| 2 | 45 | Fibroids | No | Pre-menopausal |
| 3 | 29 | Family history of EOC | No | Pre-menopausal |
| 4 | 63 | Endometrial cancer | No | Post-menopausal |
| 5 | 62 | Chronic pelvic pain | No | Post-menopausal |
| 6 | 72 | Endometrial cancer | No | Post-menopausal |
| 7 | 61 | Endometrial cancer | No | Post-menopausal |
| 8 | 64 | Endometrial cancer | No | Post-menopausal |
| 9 | 62 | Fibroids | No | Post-menopausal |

Supplementary Table 1: Clinical profile of patients donating ovaries for IHC studies on OSE.

| **Patient No** | **Age** | **Reason for surgery** | **Experimental Study** |
| --- | --- | --- | --- |
| 1 | 26 | Pelvic pain | qRT-PCR |
| 2 | 50 | Fibroids | qRT-PCR |
| 3 | 38 | Prophylactic | qRT-PCR, TLC |
| 4 | 21 | Pelvic pain | qRT-PCR, TLC |
| 5 | 26 | Prophylactic | qRT-PCR |
| 6 | 31 | Prophylactic | qRT-PCR |
| 7 | 29 | Prophylactic | qRT-PCR |
| 8 | 32 | Pelvic pain | qRT-PCR |
| 9 | 41 | Pelvic pain | qRT-PCR |
| 10 | 36 | Prophylactic | qRT-PCR |
| 11 | 48 | Fibroids | qRT-PCR |
| 12 | 49 | Fibroids | qRT-PCR |
| 13 | 29 | HMB | qRT-PCR |
| 14 | 45 | Fibroids | qRT-PCR |
| 15 | 43 | Fibroids | qRT-PCR |
| 16 | 29 | Diagnostic laparoscopy | qRT-PCR |
| 17 | 34 | Laparoscopic sterilisation | qRT-PCR |
| 22 | 29 | Pelvic pain | TLC |
| 23 | 27 | Prophylactic | TLC |
| 24 | 43 | HMB and pelvic pain | TLC |
| 25 | 41 | Hydrosalpinx | TLC |
| 26 | 33 | Prophylactic | TLC |
| 27 | 34 | Prophylactic | TLC |
| 28 | 19 | Left ovarian cyst | TLC |
| 29 | 31 | Prophylactic | TLC |
|  |  |  |  |
|  |  |  |  |
|  |  |  |  |
|  |  |  |  |

Supplementary Table 2. Clinical profile of patients donating OSE used for qRT-PCR and TLC.

| Patient number | Age | Pathological diagnosis | FIGO stage | Experimental study |
| --- | --- | --- | --- | --- |
| 1 | 69 | High grade serous carcinoma | IIIc | qRT-PCR |
| 2 | 61 | High grade serous carcinoma | IIIc | qRT-PCR |
| 3 | 53 | High grade serous carcinoma | IIIc | qRT-PCR, IHC |
| 4 | 43 | Mixed high grade serous/ endometrioid carcinoma | IIc | qRT-PCR, IHC |
| 5 | 55 | Serous borderline tumor | IIIA | qRT-PCR |
| 6 | 59 | High grade serous carcinoma | IIIc | qRT-PCR |
| 7 | 67 | High grade serous carcinoma | IIIc | qRT-PCR, IHC |
| 8 | 59 | High grade serous carcinoma | IV | qRT-PCR, IHC |
| 9 | 73 | High grade serous carcinoma | IV | qRT-PCR |
| 10 | 66 | Endometrioid carcinoma Grade 3 | IIIc | qRT-PCR, IHC |
| 11 | 88 | Mixed high grade serous / endometrioid carcinoma | IIIb | IHC |
| 12 | 61 | Endometrioid carcinoma Grade 1 | Ic | IHC |

Supplementary Table 3: EOC Patient information for IHC and qRT-PCR studies.
